# Relating Built Environment to Physical Activity: Two Failures to Validate

**DOI:** 10.3390/ijerph110201233

**Published:** 2014-01-23

**Authors:** Donald Schopflocher, Eric VanSpronsen, Candace I. J. Nykiforuk

**Affiliations:** 1 Faculty of Nursing, University of Alberta, Edmonton, AB T6G 1C9, Canada; E-Mail: donald.schopflocher@ualberta.ca; 2 Centre for Health Promotion Studies, School of Public Health, University of Alberta, 11405-87 Avenue, Edmonton, AB T6G 1C9, Canada; E-Mail: eric.vanspronsen@ualberta.ca

**Keywords:** built environment, health, Irvine Minnesota Inventory, scales, replication, reliability

## Abstract

The Irvine-Minnesota Inventory (IMI) is an audit tool used to record properties of built environments. It was designed to explore the relationships between environmental features and physical activity. As published, the IMI does not provide scoring to support this use. Two papers have since been published recommending methods to form scales from IMI items. This study examined these scoring procedures in new settings. IMI data were collected in two urban settings in Alberta in 2008. Scale scores were calculated using the methods presented in previous papers and used to test whether the relationships between IMI scales and walking behaviors were consistent with previously reported results. The scales from previous work did not show expected relationships with walking behavior. The scale construction techniques from previous work were repeated but scales formed in this way showed little similarity to previous scales. The IMI has great potential to contribute to understanding relationships between built environment and physical activity. However, constructing reliable and valid scales from IMI items will require further research.

## 1. Introduction

Obesity and related chronic diseases are recognized as a global population health challenge [1,2]. Calls have been made to move beyond behavioral risk factors such as physical activity levels for these conditions to consider associated environmental, political, economic, and social determinants in populations [3,4], including the effects of built environment features on energy balance behaviors and active living [5]. Any call to develop and implement public health and urban planning policies to address these relationships [6,7] may be premature, however. Some built environment features (e.g., land use mix, intersection density, and recreational facilities) have been shown to be related to some individual health outcomes (e.g., body mass index, physical activity, healthy eating) [8,9,10,11,12], although not consistently [13,14,15,16].

A recent review of tools used to assess environments for physical activity [15] distinguishes two general types: (1) self-reports of *perceptions of the environment* typically completed by participants in studies that also record information about physical activity information; and (2) instruments that are used to independently assess the environment for particular properties. This second category includes both *GIS-based measures* which use existing administrative data as a basis for the formation of measures, and *observational measures* or community audits that involve the direct observation of features of the built environment thought to be relevant to physical activity. The reviewers call for additional research with all tools to establish relationships between characteristics of the built environment and physical activity, as well as the relationships between different ways of assessing the built environment. Such research is important to establishing an evidence base to support specific improvements to the built environment that will result in improvements in active living and physical activity.

Our work has focused upon the objective assessment of the micro features of the built environment, in part because we wished to form a database of this information for future use by community partners. Databases of the spatial distribution of individual features of the built environment have many potential uses, including the secondary identification of specific local barriers to, and assets for, physical activity as part of community planning [17,18,19]. We chose to use the Irvine Minnesota Inventory (IMI) [20,21] because it is the most comprehensive of the community audit tools.

Two recent papers [22,23] have reported relationships between individual health behaviors and scales developed by aggregating IMI items. This paper reports our unsuccessful attempts to (1) replicate these findings using similar measures in a two different locations; and (2) our subsequent attempt to examine the properties of the proposed scales and to repeat the scale construction procedures. Our findings call into question the viability of these procedures to create scales that will generalize. We believe that it is important to make these failures known for several reasons: (1) recent work suggests that current publication practices result in many false positive findings being reported [24]; (2) the failure to publish failures to replicate introduces publication bias that will interfere with the ability of future reviews and meta-analyses to accurately summarize evidence [25,26]; and (3), the discovery of fraudulent published findings in psychology have led to an increased call to conduct and publish research which repeats and extends [27].

### 1.1. Irvine Minnesota Inventory

The IMI is a comprehensive measure of macro- and micro-level built environment characteristics thought to be linked to physical activity [20]. Trained observers rate 162 built environment characteristics for each road segment (two facing sides of one street block) in a study area. A high degree of inter-rater reliability for these ratings has been demonstrated [21]. While the developers created items that sampled four general domains (Accessibility, Pleasurability, Perceived Safety from Traffic, and Perceived Safety from Crime), no scoring procedures were initially provided to reduce or summarize the large amount of information collected by this procedure.

### 1.2. Proposed IMI Scales Concerning Physical Activity and Walking (Boarnet et al.)

Boarnet’s team [22] proposed scales of IMI items based on their associations with different physical activity measures. The Twin Cities Walking Study [23] structured the data collection. Thirty-six urban study areas, each measuring 805 m by 805 m, were selected to fit four combinations of residential density and street pattern (high density/small blocks; low density/small blocks; high density/large blocks; and low density/large blocks).

Health data for a 7 day period were obtained from 716 recruited participants (20 living in each of the study areas with a small number who did not complete the study or who had missing data). The health measures included two measures of Total Physical Activity (one obtained through accelerometer data measuring distance walked over the seven day period and one obtained by completion of the self-report International Physical Activity Questionnaire (IPAQ) [28]) and two measures of each of Total Walking, Total Walking for Leisure and Total Walking for Travel (all obtained from the self-report IPAQ and separately from a self-report travel diary).

IMI data were collected by separate observers from a random sample of 20% of the segments in each of the 36 study areas. For each of the 716 participants who supplied health data, environment measures were the means across all observed segments in the study area in which that participant lived for each of the 162 IMI items.

Analysis consisted of examining the relationships between the health measures and the built environment measures. Each IMI item was entered into a separate regression analysis to determine its relationship with each of the physical activity measures (a maximum of 9 times 162 separate regressions if all items had variability). Each regression analysis also included the covariates enumerated in Table 1.

The authors proposed that the items showing significant relationships (defined as *p* < 0.1) in these analyses could be assembled into separate scales to score the propensity of environments to support each of: Physical Activity, Total Walking, Total Walking for Travel, and Total Walking for Leisure. Two versions of scales for each of these outcome variables were proposed: a moderate version which included items associated with either the IPAQ or the travel diary version of each measure, and a prudent version restricted to the items associated with both the IPAQ and the travel diary version of the measure. An additional scale was proposed out of items associated with accelerometer data. No item weights were provided for assembling items into scales.

**Table 1:** Comparison of covariates used in the current study and Boarnet *et al.*, [22].

Covariate	Current Study	Boarnet *et al.*
**Age**	In Years	In Years
**Age squared**	In years Squared	In Years Squared
**Children**	Dummy Variable = 1 if children < 18 years in household, else 0	Dummy Variable = 1, if children < 18 years in household, else 0
**Married**	Dummy variable = 1 if married, else 0	Dummy variable = 1 if married, else 0
**Education**	Dummy variable = 1 indicating that the respondent has completed some college/university, college/university degree, or graduate/professional degree, else 0	Three dummy variables indicating highest level of education (some college, college degree, or graduate/professional degree)
**Employment**	Dummy variable = 1 if currently employed, else 0	Dummy variable = 1 if currently employed, else 0
**Student**	Dummy variable = 1 if a student, else 0	Dummy variable = 1 if a student, else 0
**Household Income**	Dummy variable = 1, indicating the respondent (and their family) may be in straitened financial circumstances	Household income, indicated by 3 dummy variables for annual income in ranges of US$20,000 to US$50,000, US$50,000 to US$80,000, and more than US$80,000
**Race/Ethnicity**	Not Collected	Race/ethnicity, indicated by 3 dummy variables for Black, Asian, and Hispanic
**Drive to work**	Not Collected	Dummy variable indicating whether respondent drives to work
**Vehicle**	Not Collected	Dummy variable = 1, if vehicle is available to respondent
**Dog**	Not Collected	Dummy variable = 1, if dog owner

### 1.3. Proposed IMI Scales Concerning Walkability in the Context of Light Rail Transit Use (Werner et al.)

Werner *et al*. [29] studied whether individuals were more likely to use light rail transit (*i.e.*, walk to a transit stop) if they lived on a “walkable” block in Salt Lake City, Utah. Light Rail Transit (LRT) usage data were collected by survey at two times points from 51 individuals living within 0.5 miles of a new transit station. To measure walkability, independent observers completed the IMI for each segment in the study area on which a participant lived. Scales were derived from the items of the IMI. Beginning with the domains proposed by the IMI authors, Werner and colleagues divided the Accessibility domain into three new sub-domains (Density, Diversity, and Pedestrian Access), renamed the Pleasurability domain (Attractiveness), and retained the Traffic Safety and Crime Safety domains. Next, they re-categorized some items to ensure that items represented only a single domain. Then standard scores were calculated for each feature, and aggregated into scales corresponding to the six domains. This was accomplished by averaging the item standard scores. Items in each domain with no rated scores were ignored when calculating these averages. A participant’s built environment scores were the scale scores representing each of the six domains for the segment on which he or she lived.

ANCOVA analyses were conducted to determine if the derived IMI scales differentiated non-users, new users, and continuing LRT users. Positive relationships were reported between LRT usage and Diversity (*p* < 0.05), Safety from Crime (*p* < 0.05) and Residential Density (*p* < 0.1).

### 1.4. Community Health and the Built Environment (CHBE) Project

The CHBE project [30] sought to uncover opportunities in four communities in Alberta, Canada, for promoting physical activity and healthy eating by overcoming barriers in the built environment while working directly with diverse communities to act on these opportunities. The process included expert assessment of the built environment, but went beyond this activity to share this information with a Community Working Group that included representatives from each community and the CHBE research team. This Working Group then jointly planned, managed, and evaluated interventions. It was hoped that the Working Group members would disseminate their enhanced understandings through their regular roles within the community, and also generate sufficient support for the process to insure its sustainability after the research project concluded. As part of this project, built environment data was collected using an adapted version of the IMI.

Individual self-reported health survey data from a computer-assisted, random-digit-dial phone survey were made available for the current analyses by the Healthy Alberta Communities (HAC) project [31], an earlier independent study that examined the effect of a number of community interventions on community obesity rates in the same Alberta communities. This linked dataset provided an opportunity to examine the findings reported by Boarnet *et al*. [22] and the scale construction procedures reported both by Boarnet *et al*. and by Werner *et al*. [29]. The study received ethical clearance from the Health Research Ethics Board (Panel B), University of Alberta.

## 2. Experimental Section

### 2.1. Built Environment Measurement

In June 2008, three observers attended a 3-day training session on the administration of an adapted version of the IMI. The IMI remained intact, but the CHBE-modified tool [30] included additional items from the Systematic Pedestrian and Cycling Environmental Scan [32] and the Pedestrian Environment Data Scan [33]. In addition, a separate IMI rating was performed on each side of the road constituting a segment in the original IMI. Finally, all segments from all four communities were rated to provide a complete database for future use. Ratings were registered on a Motorola MC35 handheld computer running CyberTrack software (CyberTracker, v3.129, CyberTracker, Cape Town, South Africa). A GPS reading was taken at the mid-point of each segment.

**Figure 1:**
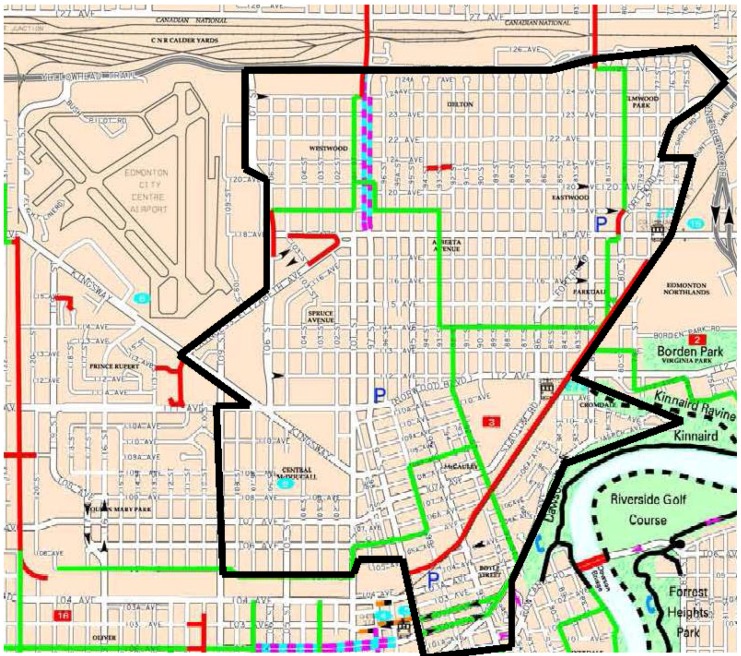
Study area for North Central Edmonton, Alberta.

**Figure 2:**
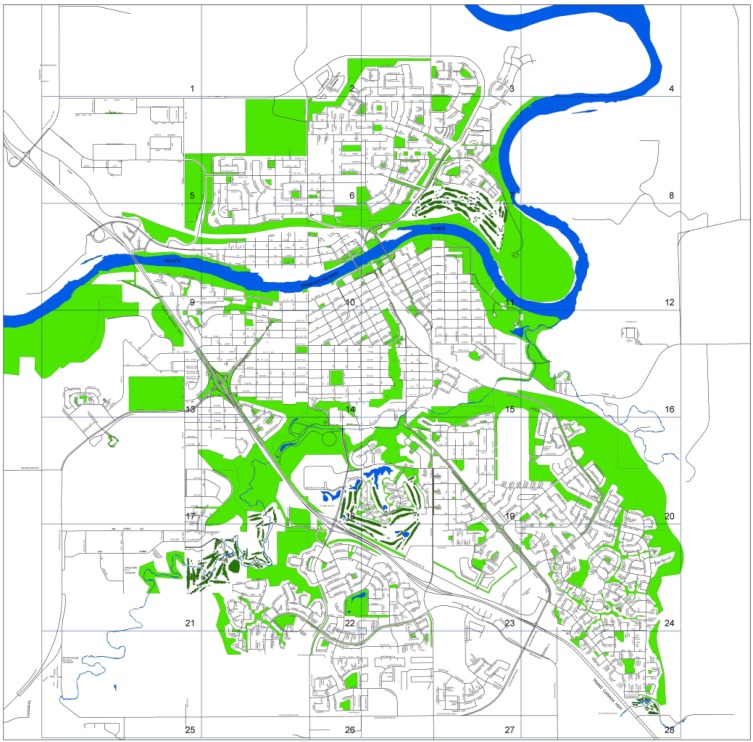
Study area for Medicine Hat, Alberta.

In total, observers documented 3,786 segments in four communities including two smaller rural communities in North East Alberta. All four communities had previously been chosen as representative sites for health promotion interventions for the Healthy Alberta Communities project [31] by government funders. Because the smaller communities differed markedly, only the data from the 3,195 segments in the two larger urban settings were used in the current work. The North Central Edmonton community (population: 41,026) [34] comprises 11 neighborhoods in Edmonton, Alberta (see Figure 1) and is characterized as an inner-city area. It is a relatively homogenous environment built on a grid pattern. Medicine Hat (population: 61,097) [35] is a city in southern Alberta that includes a wide range of built environments from downtown to suburban spaces (see Figure 2).

### 2.2. Health Survey Data

Individual self-reported health data were made available from the HAC project [31]. Measures included self-report physical activity variables for the 2,042 participants in the two urban communities examined in the current analyses (780 from North Central Edmonton and 1,262 from Medicine Hat that could be geocoded (GeoPinpoint, v6.4) to a segment within the community). (HAC participants could optionally provide an address, but not all chose to do so. Analysis suggested no differences between these groups on the physical activity measures employed here).

Physical activity questions asked in the HAC survey were taken from the Canadian Community Health Survey (CCHS) Cycle 3.1. [36,37] and had, in turn, been based on the IPAQ [28]. One difference is that CCHS data were collected for a 3-month recall period. Measures of Total Walking, Total Walking for Leisure, and Total Walking for Travel comparable to the self-report measures calculated by Boarnet *et al*. [22] could be calculated. Instructions are available for calculating Total Walking for Leisure, *i.e.*, multiplying daily frequency of walking for leisure by a categorical variable indicating the normal duration of walking for leisure. This number is then multiplied by a factor which expresses the metabolic energy cost as a multiple of the resting metabolic rate (MET). Instructions were not provided for calculating Total Walking for Travel in comparable units. As the Walking for Travel question requires a categorical response indicating the amount of time spent walking for travel in the past week, creating values for the categorical ranges of times allows for the same Total Walking for Travel variable as was calculated for Total Walking for Leisure. The following values were assigned: none = 0; <1 h = 0.5; 1–5 h = 3; 6–10 h = 8; 11–20 h = 15.5; and >20 h = 22.5. Thus, using the calculation described for Total Walking for Leisure, Total Walking for Travel was calculated by multiplying the average number of hours spent walking for travel per day by the MET value for walking. The Total Walking variable was then calculated as the sum of Total Walking for Leisure and Total Walking for Travel.

### 2.3. Statistical Analysis

All statistical analyses were conducted using SPSS v.18.0 (SPSS Inc., Chicago, IL, USA).

### 2.4. Repeating Proposed IMI Scales Concerning Physical Activity and Walking (Boarnet et al.)

**Figure 3:**
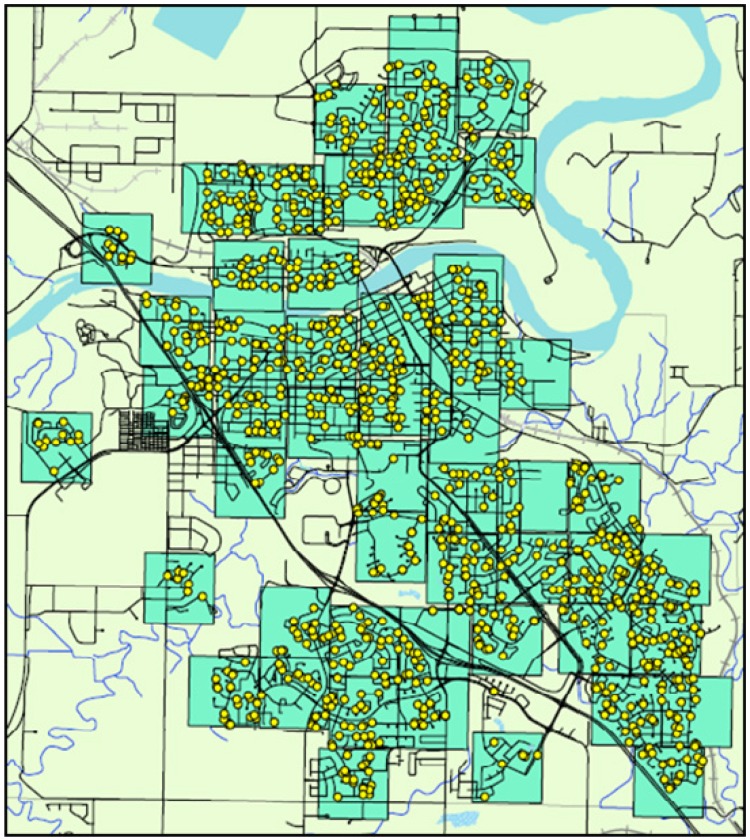
Distribution of phone survey participants and study areas across Medicine Hat, Alberta.

Data were re-coded and analyzed to approximate the methods described by Boarnet *et al*. [22]. Applying an 805 m × 805 m grid to CHBE data (ArcGIS Desktop: Release 10, Redlands, CA, USA), 50 study areas were created for Medicine Hat and 16 study areas were created for North Central Edmonton. By way of illustration Figure 3 shows the study areas and distribution of respondents for Medicine Hat.

Built environment feature scores were the average scores for the individual features across observed segments within the individual’s study area. However, 100% of the segments inside the study area were used (rather than 20%).

Analysis consisted of three phases: (1) examining the associations between the Boarnet *et al*. proposed scales and the summary Walking variables; (2) examining the internal consistency of the Boarnet *et al*. scales; and (3) repeating the Boarnet *et al*. procedures to examine whether the items for recommended scales were similar.

As accelerometer and travel diary information were not collected by HAC, only the Boarnet *et al*. scales based on self-reported physical activity survey data (*i.e.*, Total Walking, Total Walking for Leisure, or Total Walking for Travel) could be examined. Averages were taken for all IMI items proposed for the moderate scales by Boarnet *et al*. to represent each scale. Internal consistency of the items in these scales was assessed by calculating Cronbach’s alpha [38]. To repeat the Boarnet *et al*. scale construction procedures, regression analyses were performed separately in each of our two communities to determine the relationship between the three physical activity scales (Total Walking, Walking for Leisure, and Walking for Travel) and each of the IMI items as corrected for the variables in Table 1. The overlap between the scales proposed by Boarnet *et al.* and the items suggested by these analyses were examined.

### 2.5. Generalizing Proposed IMI scales Concerning Walkability in the Context of Light Rail Transit Use (Werner et al.)

While the focus of Werner *et al*. [29] was on walking to LRT stations, the current analysis applied their derived scales to the more general walking behaviors: Total Walking, Walking for Leisure, and Walking for Travel. As in the Werner *et al*. analysis, each participant’s six scale scores (Density, Diversity, Pedestrian Access, Attractiveness, Safety from Traffic, Safety from Crime) were based on the average of standardized scores on the street segment of their residence for each variable in the scale as specified by Werner *et al*. Multiple linear regression was used to determine if a relationship existed between the built environment scales and each walking behavior measure. As the walking behaviors reported here are continuous variables, multiple linear regression was used in place of ANCOVA. Analyses were corrected for age and gender, in addition to education. Finally, internal consistency of the six IMI derived scales was examined by calculating Cronbach’s alpha.

## 3. Results

### 3.1. Results of Repeating Proposed IMI Scales Concerning Physical Activity and Walking (Boarnet et al.)

Table 2 summarizes the relationships between the walking behavior scales from the HAC data and the scales calculated according to the recommendations of Boarnet *et al*. None of the correlation coefficients met conventional levels of statistical significance. These results did not change when the relationships were examined by regression analyses that corrected for the variables in Table 1.

**Table 2:** Correlations between Boarnet *et al*. scales and HAC outcome measures.

Boarnet-Derived Scales	CHBE Setting	HAC Outcome Measure *		
		Total Walking	Walking For Leisure	Walking for Travel
**“To Predict Total Walking”**	Medicine Hat	0.160	0.097	0.135
	North Central Edmonton	0.099	−0.17	0.123
**“To Predict Walking for Leisure”**	Medicine Hat	0.043	0.112	−0.008
	North Central Edmonton	0.170	0.263	0.149
**“To Predict Walking for Travel”**	Medicine Hat	−0.071	−0.110	−0.072
	North Central Edmonton	−0.128	−0.110	−0.101

* All correlation coefficients were insignificant (*p* > 0.05).

Table 3 shows the internal consistency of the Boarnet *et al*. recommended scales, as measured by Cronbach’s alpha. Values range from below 0 (which indicates that on average the items have negative correlations with each other) to 0.608. Common rules of thumb for internal consistency place the limit of acceptability for a scale at 0.7 or greater, but suggest 0.8 or higher for a scale’s routine use [39].

Table 4 summarizes how the items recommended by Boarnet *et al*. fared in the current data in predicting the scales to which they had been assigned, based on a level of significance of *p* < 0.10. Only a very few of these items show relationships here.

**Table 3:** Internal consistency of Boarnet *et al*. scales.

Boarnet-Derived Scales	CHBE Setting	Scale Reliability (Cronbach’s alpha)
**“To Predict Total Walking”**	Medicine Hat	0.608
	North Central Edmonton	<0.00
**“To Predict Walking for Leisure”**	Medicine Hat	0.246
	North Central Edmonton	0.489
**“To Predict Walking for Travel”**	Medicine Hat	0.255
	North Central Edmonton	<0.00

**Table 4:** Number of items in each Boarnet *et al.* recommended IMI moderate scale demonstrating significant associations with comparable physical activity behaviors in CHBE settings.

IMI Moderate Scale	Boarnet *et al*. Results	CHBE/HAC Results In Two Settings	
	Number of variables in scale (*p* < 0.1)	Setting	Number of Variables in Scale Demonstrating Significance (*p* < 0.1)
**Total Walking**	14	Medicine Hat	3
		North Central Edmonton	0
**Walking for Leisure**	11	Medicine Hat	1
		North Central Edmonton	1
**Walking for Travel**	17	Medicine Hat	0
		North Central Edmonton	0

Table 5 summarizes the scales that would have been constructed by replicating the procedures of Boarnet *et al*. [22]. Note the very small number of common scale items across the two CHBE settings. Most of the items that would have been included in scales for either CHBE setting were not included in Boarnet *et al*. scales.

**Table 5:** Number of IMI items that would be present in each scale for CHBE/HAC data if created using the Boarnet *et al*. procedures.

CHBE/HAC Scales	CHBE Setting	Number of Variables in Scale	Variables with *p* < 0.05	Variables with *p* < 0.1	Number of Common Variables
**Total Walking**	Medicine Hat	40	30	10	3
	North Central Edmonton	11	2	9	
**Walking for Leisure**	Medicine Hat	57	44	13	4
	North Central Edmonton	13	4	9	
**Walking for Travel**	Medicine Hat	18	8	10	3
	North Central Edmonton	17	8	9	

### 3.2. Results of Generalizing Proposed IMI Scales Concerning Walkability in the Context of Light Rail Transit Use (Werner et al.)

A comparison of the relationships between domains of the built environment and health behaviors is presented in Table 6. For the Werner results, the level of significance is reported for relationships between LRT use and the independent variables. Standardized beta coefficients for the regression analyses and level of significance are presented for results generated from CHBE/HAC data. In Medicine Hat, a statistically significant relationship was discovered between the Attractiveness scale and the Walking for Leisure outcome. In North Central Edmonton, statistically significant relationships were discovered between the Crime Safety variable and two health outcomes: Total Walking and Walking for Travel. A consistent pattern of significance was not found for any relationships between built environment domains and physical activity behaviors across all three settings. The Diversity scale is found to have a significant relationship with walking behaviors only by Werner. Crime Safety was found to have significant relationship with walking behaviors by Werner, and in CHBE’s North Central Edmonton setting. In North Central Edmonton, this relationship is negative, suggesting less Total Walking and less Walking for Travel in areas considered safer from crime. The Attractiveness scale is an indicator of any walking behaviors only in Medicine Hat.

**Table 6:** Regression analysis of built environment scales and walking behaviors in Werner and CHBE Settings.

	Medicine Hat (standardized beta coefficients)			North Central Edmonton (standardized beta coefficients)			Werner
	Total Walking	Walking for Leisure	Walking for Travel	Total Walking	Walking for Leisure	Walking for Travel	LRT Usage
**Diversity**	0.048	0.034	0.038	−0.081	−0.002	−0.085	*
**Density**	−0.030	−0.012	−0.033	−0.050	−0.047	−0.035	**
**Pedestrian Accessibility**	−0.029	0.002	−0.031	0.038	−0.050	0.068 **	
**Crime Safety**	−0.031	0.033	−0.040	−0.095 *	−0.008	−0.101 *	*
**Traffic Safety**	−0.003	0.057 **	−0.023	0.025	0.012	0.022	
**Attractiveness**	0.015	0.062 *	−0.004	−0.017	−0.065	0.009	
**Age**	−0.209	0.327 **	−0.374 *	0.096	0.410 **	−0.064	
**Age-squared**	0.146	−0.349 *	0.318 **	−0.191	−0.380	−0.061	
**Gender**	−0.057 **	−0.031	−0.058 **	−0.063	−0.022	−0.067 **	
**Higher Education**	0.029	0.002	0.030	0.032	−0.030	0.045	
**R-squared**	0.015 **	0.016 **	0.016 **	0.030 **	0.017	0.040 *	

* *p* < 0.05; ** *p* < 0.10.

Table 7 presents Cronbach alphas as indicators of the internal consistency of the scales in both CHBE settings. Again, values range from below 0 to 0.75. Differences across the domains are less marked than for the Boarnet *et al*. scales, but again these values do not reach accepted levels for the internal consistency of a scale.

**Table 7:** Internal consistency of scales informed by methods used by Werner.

Werner-Derived Scales	CHBE Setting	Scale Internal Consistency (Cronbach’s alpha)
**Diversity**	Medicine Hat	0.75
	North Central Edmonton	0.51
**Density**	Medicine Hat	<0.00
	North Central Edmonton	<0.00
**Pedestrian Accessibility**	Medicine Hat	0.23
	North Central Edmonton	0.37
**Crime Safety**	Medicine Hat	0.35
	North Central Edmonton	0.50
**Traffic Safety**	Medicine Hat	0.61
	North Central Edmonton	0.48
**Attractive**	Medicine Hat	0.43
	North Central Edmonton	0.53

## 4. Discussion

This study failed to support the hypotheses of Boarnet *et al*. [22] and failed to support the viability of scales created using the methods of either Boarnet *et al*. [22] or Werner *et al*. [29] in the current settings. We can offer no evidence that the scales produced by either research group have general value for establishing relationships between built environment features and health behaviors. The reasons relate not only to the lack of consistent relationships between scales derived from the IMI and physical activity measures, but also to general properties of the scales derived by the procedures used by Boarnet *et al*. and by Werner *et al*.

It is possible that two or more items, each with very little relationship to each other, may both contribute to the prediction of another variable (such as a health behavior) and therefore may conceivably be combined into a scale to use to predict that other variable. The method typically used to establish such a scale would be multiple regression analysis. However, multiple regression analyses generally require large samples in order to be reliable in this task. Rules of thumb for the number of individuals required per item considered range from a low of 10 [40] to 30 or higher [41]. In Boarnet *et al*.’s [22] analysis, there is a sample of 716 persons on at least 178 IMI items, considerably below what would be considered sufficient to produce replicable findings. However, the situation is much poorer than even this sample size would suggest. Because there were only 36 study areas over which built environment scores were derived by averaging, there were only 36 possible sets of scores for the 716 participants on those environment scales, and as a result, the sample size is effectively only 36. This very small effective sample size helps to explain why Boarnet *et al*. needed to conduct their analyses one IMI variable at a time rather than including many items simultaneously in their regression analyses. However, this has resulted in a very large number of analyses, each of which is reported at a “relaxed” level of statistical significance. For a large number of such tests (*i.e.*, the multiple comparison problem), conventional wisdom is that significance levels should be substantially tightened rather than relaxed [42]. Altogether it should come as no shock that the scales suggested by Boarnet *et al*. perform poorly in another setting.

In addition, all of the proposed scales examined here showed low alpha coefficients. That is, the collections of items that are aggregated into single scales do not intercorrelate very highly, and therefore there is minimal evidence that they are measuring anything in common. The scales proposed by Werner *et al*. [29] combine items classified into domains related to particular abstract properties of the built environment. If such domain classifications were accurate, then the items that measure a single property should intercorrelate, and, in turn, aggregating them into scales should result in scales with high(er) internal consistency coefficients. Werner *et al*. do not calculate internal consistency coefficients. However, it would have been possible for Werner *et al*. to report alpha coefficients even though there were many items that could not be scored for particular segments. In fact the scoring procedure that they used (for each individual, averaging the standard scores for items which did have a score) is formally equivalent to assigning that mean to each item that could not be scored. When this is explicitly done, alpha coefficients can be calculated as we have demonstrated above.

These low alpha coefficients suggest that the properties in the proposed classifications of items are not well represented by these scales. This may be because of a very large number of very narrow items, and a small number of very broad concepts. One way to proceed in attempting to develop scales for the IMI would be to look for a larger number of intermediate properties within each domain and seek items that intercorrelate with each other to form into scales. If such can be discovered, they would have high(er) internal consistency and should be easier to validate. For example, preliminary work suggests that IMI items indicating the presence of green spaces are associated with the presence of schools, traffic calming devices, and controlled crossings. Such a cluster of properties might in turn be associated with greater physical activity among individuals living close to it. Our team is currently engaged in attempting to form such scales from the items of the IMI using methodologies that have long been used for this purpose in psychometrics [42].

For policy and planning activities, successful scales would be able to locate clusters of interlocking properties or their absence that might support particular outcomes. This does not mean that the potential of individual items such as locating precisely the location of particular environmental features would be lost; rather an appropriate contextualization of such specific features might be encouraged in efforts to establish successful policies to modify the built environment.

### Strengths and Limitations

The current study was based upon a substantially larger data set than either the Boarnet *et al*. or the Werner *et al*. studies. It included IMI data on all segments rather than a sample of segments and thus avoided sampling error in the environmental ratings. It also included information from two differing urban environments which together provided a larger number of study areas than were available in the Boarnet *et al*. study.

However, the current study did not have exactly comparable physical activity outcome measures to the Boarnet *et al*. or the Werner *et al*. studies. Thus the current study did not have direct behavioral (accelerometer) data as the Boarnet *et al*. study did. As result, we were unable to examine whether the specific scales that Boarnet *et al*. formed to predict this data were replicable. However, we were clearly unable to replicate the recommended Boarnet *et al*. scales to predict self-report walking data, even where our measures differed primarily in the span of time over which the self-report measures were reported. Similarly, we did not have an outcome variable comparable to the LRT usage variable in Werner *et al*. study, and it remains possible that their procedures would work for their outcome variable in future studies. Nevertheless since the scales that they propose were general scales that together include the majority of IMI items, we believe that they should show internal consistency as measured by coefficient alpha, and that they should be sufficiently broad to also correlate with related outcome variables such as the ones used here. This property of scales is generally taken to be an important part of the external validity of the underlying concept putatively being measured by the scale [43].

## 5. Conclusions

By failing to validate the findings or procedures of other research groups, this paper demonstrated that no acceptable scoring scheme has yet been developed for the IMI. The authors are currently applying psychometric methods to create such a scoring scheme. Until a better scoring scheme is available, any generalizations concerning the impact of the built environment on population health using the IMI tool remain premature.

## References

[1] Finucane M.M., Stevens G.A., Cowan M.J., Danaei G., Lin J.K., Paciorek C.J., Singh G.M., Gutierrez H.R., Lu Y., Bahalim A.N. (2011). National, regional, and global trends in body-mass index since 1980: Systematic analysis of health examination surveys and epidemiological studies with 960 country-years and 9.1 million participants. Lancet.

[2] Swinburn B.A., Sacks G., Hall K.D., McPherson K., Finegood D.T., Moodie M.L., Gortmaker S.L. (2011). The global obesity pandemic: Shaped by global drivers and local environments. Lancet.

[3] Raine K.D. (2004). Obesity and Overweight in Canada: A Population Health Perspective.

[4] World Health Organization: Integrated Chronic Disease Prevention and Control. World Health Organization. http://www.who.int/chp/about/integrated_cd/en/.

[5] Egger G., Swinburn B. (1997). An ecological approach to the obesity pandemic. BMJ.

[6] Frank L., Kavage S. (2009). A national plan for physical activity: The enabling role of the built environment. J. Phys. Act. Health.

[7] Heath G.W., Brownson R.C., Kruger J., Miles R., Powell K.E., Ramsey L.T. (2006). The effectiveness of urban design and land use and transport policies and practices to increase physical activity: A systematic review. J. Phys. Act. Health.

[8] Adams M.A., Sallis J.F., Kerr J., Conway T.L., Saelens B.E., Frank L.D., Norman G.J., Cain K.L. (2011). Neighborhood environment profiles related to physical activity and weight status: A latent profile analysis. Prev. Med..

[9] Garden F.L., Jalaludin B.B. (2008). Impact of urban sprawl on overweight, obesity, and physical activity in Sydney, Australia. J. Urban Health.

[10] Hoehner C.M., Handy S.L., Yan Y., Blair S.N., Berrigan D. (2011). Association between neighborhood walkability, cardiorespiratory fitness and body-mass index. Soc. Sci. Med..

[11] Joshu C.E., Boehmer T.K., Brownson R.C., Ewing R. (2008). Personal, neighbourhood and urban factors associated with obesity in the United States. J. Epidemiol. Community Health.

[12] Frank L.D., Saelens B.E., Powell K.E., Chapman J.E. (2007). Stepping towards causation: Do built environments or neighborhood and travel preferences explain physical activity, driving, and obesity?. So. Sci. Med..

[13] Ding D., Sallis J.F., Kerr J., Lee S., Rosenberg D.E. (2011). Neighborhood environment and physical activity among youth: A review. Am. J. Prev. Med..

[14] Feng J., Glass G.A., Curriero F.C., Stewart W.F., Schwartz B.S. (2010). The built environment and obesity: A systematic review of the epidemiologic evidence. Health Place.

[15] Brownson R.C., Hoehner C.M., Day K., Forsyth A., Sallis J.F. (2009). Measuring the built environment for physical activity state of science. Am. J. Prev. Med..

[16] Wendel-Vos W., Droomers M., Kremers S., Brug J., van Lenthe F. (2007). Potential environmental determinants of physical activity in adults: A systematic review. Obes. Rev..

[17] Deehr R.C., Shumann A. (2009). Active Seattle: Achieving walkability in diverse neighborhoods. Am. J. Prev. Med..

[18] Bors P.A., Brownson R.C., Brennan L.K. (2012). Assessment for active living: Harnessing the power of data-driven planning and action. Am. J. Prev. Med..

[19] Evenson K.R., Sallis J.F., Handy S.L., Bell R., Brennan L.K. (2012). Evaluation of physical projects and policies from the active living by design partnerships. Am. J. Prev. Med..

[20] Day K., Boarnet M., Alfonzo M., Forsyth A. (2006). The Irvine-Minnesota inventory to measure built environments: Development. Am. J. Prev. Med..

[21] Boarnet M.G., Day K., Alfonzo M., Forsyth A., Oakes M. (2006). The Irvine-Minnesota inventory to measure built environments: Reliability tests. Am. J. Prev. Med..

[22] Boarnet M.G., Forsyth A., Day K., Oakes J.M. (2011). The street level built environment and physical activity and walking: Results of a predictive validity study for the Irvine Minnesota Inventory. Environ. Behav..

[23] Oakes J.M., Forsyth A., Hearst M.O., Schmitz K.H. (2009). Recruiting participants for neighbourhood effects research: Strategies and outcomes of the Twin Cities Walking Study. Environ. Behav..

[24] Ioannidis J.P. (2005). Why most published research findings are false. PLoS Med..

[25] Dickersin K., Min Y.I. (2006). Publication bias: The problem that won’t go away. Ann. N. Y. Acad. Sci..

[26] Dwan K., Altman D.G., Arnaiz J.A., Bloom J., Chan A.W., Cronin E., Decullier E., Easterbrook P.J., von Elm E., Gamble C. (2008). Systematic review of the empirical evidence of study publication bias and outcome reporting bias. PLoS One.

[27] Roediger H.L. (2012). Psychology’s woes and a partial cure: The value of replication. APS Obs..

[28] Craig C.L., Marshall A.L., Sjostrom M., Bauman A.E., Booth M.L., Ainsworth B.E., Pratt M., Ekelund U., Yngve A., Sallis J.F. (2003). International physical activity questionnaire: 12-country reliability and validity. Med. Sci. Sports Exerc..

[29] Werner C.N., Brown B.B., Gallimore J. (2010). Light rail use is more likely on “walkable” blocks: Further support for using micro-level environmental audit measures. J. Environ. Psychol..

[30] Nykiforuk C.I.J., Schopflocher D., Vallianatos H., Spence J.C., Raine K.D., Plotnikoff R.C., Vanspronsen E., Nieuwendyk L. (2013). Community health and the built environment: Examining place in a Canadian chronic disease prevention project. Health Promot. Int..

[31] Raine K.D., Plotnikoff R., Nykiforuk C., Deegan H., Hemphill E., Storey K., Schopflocher D., Veugelers P., Wild T.C., Ohinmaa A. (2010). Reflections on community-based population health intervention and evaluation for obesity and chronic disease prevention: The healthy Alberta communities project. Int. J. Public Health.

[32] Pikora T.J., Bull F.C.L., Jamrozik K., Knuiman M., Giles-Corti B., Donovan R.J. (2002). Developing a reliable audit instrument to measure the physical environment for physical activity. Am. J. Prev. Med..

[33] Clifton K.J., Smith A.D.L., Rodriguez D. (2007). The development and testing of an audit for the pedestrian environment. Landsc. Urban Plan..

[34] The City of Edmonton (2009). 2009 Municipal Census Results. http://www.edmonton.ca/city_government/2009-municipal-census-results.aspx.

[35] City of Medicine Hat (2009). City of Medicine Hat 2009 Census Final Report. http://www.medicinehat.ca/modules/showdocument.aspx?documentid=276.

[36] Statistics Canada (2006). Canadian Community Health Survey (CCHS) Cycle 3.1 (2005) Public Use Microdata File (PUMF) User Guide. http://www23.statcan.gc.ca:81/imdb-bmdi/pub/document/3226_D7_T9_V3-eng.pdf.

[37] Statistics Canada (2006). Canadian Community Health Survey (CCHS) Cycle 3.1 (2005) Public Use Microdata File (PUMF) Integrated Derived Variable and Grouped Variable Specifications. http://www23.statcan.gc.ca:81/imdb-bmdi/pub/document/3226_D7_T9_V3-eng.pdf.

[38] Cronbach L.J. (1951). Coefficient alpha and the internal structure of tests. Psychometrika.

[39] Kline P. (1999). The Handbook of Psychological Testing.

[40] Aguinis H., Harden E.E., Lance C.E., Vandenberg R.J. (2008). Sample Size Rules of Thumb: Evaluating Three Common Practices. Statistical and Methodological Myths and Urban Legends: Doctrine, Verity and Fable in Organizational and Social Science.

[41] VanVoorhis C.R.W., Morgan B.L. (2007). Understanding power and rules of thumb for determining sample sizes. Tutor. Quant. Methods Psychol..

[42] Curran-Everett D. (2000). Multiple comparisons: Philosophies and illustrations. Am. J. Physiol. Regul. Integr. Comp. Physiol..

[43] Nunnally J., Bernstein I. (1994). Psychometric Theory.

